# Effect of antiretroviral therapy on gut microbiota, immune activation, and inflammatory markers in newly diagnosed HIV patients

**DOI:** 10.3389/fcimb.2026.1853799

**Published:** 2026-07-27

**Authors:** Denisse Márquez-Ruiz, Francisco Illanes-Álvarez, Andrés Martín-Aspas, Ismael Tinoco Racero, Irene Campaña-Gómez, Raquel Guerra-González, Alberto Romero-Palacios, Mercedes Márquez-Coello, Sara Cuesta-Sancho, José-Antonio Girón-González

**Affiliations:** 1 Servicio de Medicina Interna, Unidad de Enfermedades Infecciosas, Hospital Universitario Puerta del Mar, Cádiz, Spain; 2 Instituto para la Investigación e Innovación Biomédica de Cádiz (INiBICA), Cádiz, Spain; 3 Facultad de Medicina, Universidad de Cádiz, Cádiz, Spain; 4 Unidad de Enfermedades Infecciosas, Facultad de Medicina, Hospital Universitario Puerto Real, Cádiz, Spain; 5 Mucosal Immunology Lab, Department of Paediatrics and Immunology, University of Valladolid, Institute of Biomedicine and Molecular Genetics (IBGM, University of Valladolid-CSIC), Valladolid, Spain; 6 Health Research Institute of Valladolid (IBioVALL), Valladolid, Spain

**Keywords:** antiretroviral therapy, gut, HIV, immune activation, inflammation, microbiota

## Abstract

**Introduction:**

People living with HIV (PLWH) exhibit gut dysbiosis and chronic immune activation. The early dynamics of the gut-immune axis during the transition from diagnosis to viral suppression remain incompletely understood.

**Methods:**

We characterized fecal microbiota (16S rRNA), bacterial translocation (16S rDNA) and gut barrier integrity (serum intestinal fatty acid-binding protein, I-FABP, level) in newly diagnosed PLWH at baseline (t0) and after six months of suppressive ART (t6), compared to uninfected controls. Systemic inflammation (serum concentrations of interleukins 6 and 10, soluble CD163), and peripheral blood T-cell activation were also analyzed.

**Results:**

Alpha diversity was similar across groups (p>0.05); beta diversity differed significantly between PLWH at t0 and controls (p=0.002). Linear Discriminant Analysis Effect Size (LEfSe) analysis identified an enrichment of short chain fatty acids (SCFA)-producing genera (*Alloprevotella*, *Mitsuokella*, *Succinivibrio*, *Megasphaera*) in PLWH at t0. PLWH at t0 showed elevated bacterial translocation [16S rDNA, 4281 (4018-4485) vs 2162 (2023-2286) copies/mL, p<0.001], gut barrier damage [I-FABP, 950 (800-1168) vs 767 (574-902) ng/mL, p=0.012], inflammation [IL-6, 4.8 (3.1-8.8) vs 0.8 (0.3-1.3) pg/mL, p<0.001; sCD163, 1173 (872-1417) vs 561 (425-662) ng/mL, p<0.001], and T-cell activation [CD4^+^CD38^+^HLA-DR^+^ (percentage of CD4^+^ T lymphocytes), 9.7 (2.8-18.5) vs 1.3 (0.3-2.2), p=0.007; CD8^+^CD38^+^HLA-DR^+^ (percentage of CD8+T lymphocytes), 19.0 (12.0-31.1) vs 1.7 (0.6-5.7), p=0.005], compared with controls. Treated PLWH (t6) showed similar alpha and beta diversities of fecal microbiota compared to untreated PLWH (p>0.05 in each case). LEfSe analysis identified an enrichment of *Campylobacter* and *Atopobium* at t6. ART mitigates systemic inflammation [t6 vs t0, IL-6, 3.3 (1.7-4.0) vs 4.8 (3.1-8.8) pg/mL, p=0.022; sCD163, 795 (549-915) vs 1173 (872-1417) ng/mL, p<0.001], and T-cell activation [t6 vs t0, CD4^+^CD38^+^HLA-DR^+^ (percentage of CD4^+^ T lymphocytes), 2.7 (1.7-6.3) vs 9.7 (2.8-18.5), p=0.047; CD8^+^CD38^+^HLA-DR^+^ (percentage of CD8^+^T lymphocytes), 6.5 (3.7-15.5) vs 19.0 (12.0-31.1), p=0.086].

**Conclusions:**

Untreated PLWH show a dysbiotic signature, characterized by enrichment of SCFA-producers. Compared to controls, the fecal microbiota of PLWH after 6 months of ART (t6) continued to show differences characterized by a predominance of *Alloprevotella* and *Succinivibrio*, among others, and continued elevations in intestinal injury, bacterial translocation, and inflammatory/immune activation markers, underscoring the incomplete normalization of gut-immune homeostasis.

## Introduction

People living with human immunodeficiency virus (HIV) (PLWH) exhibit chronic immune activation, which contributes significantly to comorbidities (Márquez-Coello et al., 2019; Zicari et al., 2019). A key factor in this process is microbial translocation, whereby bacterial products cross a compromised intestinal barrier and enter systemic circulation (Brenchley et al., 2006). This phenomenon triggers sustained inflammation and immune activation (Sandler and Douek, 2012; Hunt et al., 2014), perpetuating a vicious cycle that drives HIV pathogenesis.

Intestinal dysbiosis, characterized by differences in gut microbial communities, is commonly observed in PLWH (Nganou-Makamdop and Douek, 2024). Studies have described a reduction of beneficial commensals such as *Lachnospiraceae* and *Ruminococcaceae*, alongside an expansion of potentially pathogenic taxa, notably within the *Gammaproteobacteria* class and *Enterobacteriales* order (Dillon et al., 2014). The degree of dysbiosis is often correlated with immunologic status, particularly CD4^+^ T cell counts (Monaco et al., 2016; Vujkovic-Cvijin et al., 2020), suggesting that immune depletion and microbial imbalance are tightly linked.

Moreover, gut microbiota composition critically influences intestinal barrier integrity, necessary to prevent bacterial translocation. Beneficial taxa promote Th17 lymphocyte differentiation, essential for mucosal barrier maintenance (Planas et al., 2019), enhance mucus thickness (*Akkermansia muciniphila*), and produce short chain fatty acids (SCFAs) (Lukovac et al., 2014). SCFAs exert anti-inflammatory effects, serve as the primary energy source for colonocytes, and strengthen epithelial integrity and mucosal immunity (Mathewson et al., 2016; Yang et al., 2020). Conversely, pathogens such as hydrogen sulfide-producing genera exert toxic effects on colonocytes (Hunt, 2010).

While several cross-sectional studies have characterized microbiota and immunologic alterations in chronically HIV-infected individuals, there is limited longitudinal data focusing on patients at the earliest stages of infection and after initial antiretroviral therapy (ART). The objective of this study was to longitudinally analyze a cohort of newly diagnosed HIV-infected individuals at baseline (pre-treatment) and after six months of ART, when viral replication was controlled. An age- and sex-matched group of HIV-uninfected individuals was included as controls. We aimed to characterize gut microbiota diversity and composition, focusing on differences between groups and changes over time. In addition, we measured markers of bacterial translocation, and systemic inflammation, and performed immune phenotyping of CD4^+^ and CD8^+^ subsets. Finally, we explored correlations between specific microbial taxa and these immunologic and inflammatory parameters.

Our approach provides novel insights into early gut microbiota alterations and their association with immune and inflammatory responses in HIV infection, which could highlight potential targets for therapeutic modulation in the context of ART.

## Materials and methods

### Study design

This prospective, observational study included consecutive cases of newly diagnosed HIV infection recruited from a cohort of patients followed longitudinally in the HIV outpatient clinics at Puerta del Mar University Hospital (Cádiz, Spain).

Seventeen PLWH were included. These were newly diagnosed patients who started ART. They were studied at baseline (t0) and again after six months of ART (t6), at which point viral load was suppressed. Additionally, 18 age- and sex-matched HIV-uninfected individuals were recruited as controls. All participants provided written informed consent prior to their involvement. This study was approved by the Comité Coordinador de Ética de la Investigación Biomédica de Andalucía (Spain) (Reference code: SICEIA-2024-000270).

The exclusion criteria were: 1) presence of opportunistic infections or neoplasms; our screening procedure followed the guidelines established by the Spanish Group for AIDS Study (GESIDA, 2025); 2) active drug use (cocaine, heroin, amphetamines) or significant alcohol ingestion (greater than 50 g/day); 3) antibiotics or treatments that could have modified the determination of intestinal microbiota or inflammation-related molecules or cells (anti-inflammatory or immunosuppressive drugs) in the last 3 months; 4) red blood cell or plasma transfusion in the month before inclusion; 5) non-acceptance of follow-up.

### Definitions

Viral suppression was considered if the HIV load was less than 50 copies/ml (Abbott RealTime HIV-1, Abbott Park, IL, USA).

Increased serum concentration of intestinal fatty acid-binding protein (I-FABP) was indicative of gut barrier disruption (Ancona et al., 2021). The bacterial translocation was detected by serum 16S ribosomal DNA (16S rDNA) levels (Abad-Fernández et al., 2013).

Serum concentration of interleukin (IL)-6 and soluble CD163 (sCD163) were used as markers of inflammatory activation. IL-10 was measured as an anti-inflammatory marker. The activated CD4^+^ and CD8^+^ T lymphocytes were defined by the simultaneous expression of HLA-DR and CD38 on their surface.

### Study samples and participants

The study protocol included: 1) collection of clinical history, nadir CD4^+^ T cell counts, and HIV viral load; 2) stool sample collection for fecal microbiota analysis; 3) peripheral blood sampling at inclusion to assess inflammatory markers, bacterial translocation, and immune cell phenotyping.

PLWH initiated ART according to the guidelines of the Spanish Group for AIDS Study (GESIDA, 2025) and were followed for six months, at which point the same samples were collected again.

Comparisons were made between HIV-uninfected controls and patients at baseline (t0), as well as between the results from PLWH at t0 and after six months of ART (t6).

### Laboratory methods

#### Blood collection and processing

Blood samples were obtained by peripheral venipuncture. Serum was obtained by centrifugation at 2500 g during 15 min at 4 °C of blood samples in pyrogen-free heparinized tubes (Biofreeze, Costar, USA), and it was subsequently frozen down at -80 °C until its use. Quantikine Human Immunoassays (R&D, Minneapolis, MN, USA) were used to quantify serum concentrations of I-FABP, IL-6, sCD163, and IL-10, following the indications of the manufacturer.

16S rDNA in blood was detected after an initial extraction of DNA (QIAamp DNA Mini Kit; QIAgen, Hilden, Germany) and quantification by spectrophotometry (BioRad, Hercules, CA, United States), as previously described (Márquez-Coello et al, 2021).

Fresh blood samples from pyrogen-free heparinized tubes with EDTA (Biofreeze, Costar, United States) were used for flow cytometry. Activated CD4^+^ and CD8^+^ T lymphocytes (CD4^+^ and CD8^+^ CD38^+^HLA-DR^+^) were identified using a multi-step gating strategy. Lymphocytes were selected based on their characteristic forward (FSC-A) and side (SSC-A) scatter. Then, the CD3^+^ population was selected (clone SK7, BD Biosciences, San Jose, CA, United States), and it was further divided into CD4^+^ (clone SK3, BD Biosciences, San Jose, CA, United States) and CD8^+^ (clone SK1, BD Biosciences, San Jose, CA, United States) populations. Finally, activated T cells were defined within each CD4^+^ and CD8^+^ subset by the co-expression of activation markers CD38^+^ (clone HB-7, BD Biosciences, San Jose, CA, United States) and HLA-DR+ (clone L243, BD Biosciences, San Jose, CA, United States). The stained cells were acquired and analyzed in a BD FACSCanto™ II Cell cytometer, using BD FACSDiva™ Software (BD Biosciences, San Jose, CA, United States). In each case, dead cells were excluded and 300,000 cells were acquired. Blank tubes with no antibodies were used to confirm the specificity of the staining and discriminate the sample from the background.

#### Fecal sample collection and processing

Feces were collected and dissolved in tubes with preservative liquids (Stool Sample Collection & Stabilization kit, Canvax, Valladolid, Spain) that were stored at -80 °C until their use. Total genomic DNA was isolated using the QIAamp^®^ DNA Stool Mini Kit (Qiagen, Düsseldorf, Germany) according to the manufacturer’s instructions and its concentration was then determined by a Qubit fluorimeter and the Qubit™ dsDNA HS Assay Kit (Invitrogen, Thermo Scientific, Waltham, MA, USA). The amplicon was designed with an insert size that allows the readings of each pair to overlap, generating an assembled read (for each pair) that covers both variable regions V3 and V4 (Yacoub et al., 2017). The samples were amplified following the protocol instructions from Illumina (Illumina, INC, San Diego, CA, USA) (16S Metagenomic Sequencing Library Preparation). Sequencing was performed using the Illumina MiSeq Instrument (Illumina, INC, San Diego, CA, USA) with two reads for every 300 base pairs, utilizing the forward primer 5’-TCGTCGGCAGCGTCAGATGTGTATAAGAGACAGCCTACGGGNGGCWGCAG-3’ and reverse primer 5’-GTCTCGTGGGCTCGGAGATGTGTATAAGAGACAGGACTACHVGGGTATCTAATCC-3’.

### Statistical and bioinformatic analyses

Data were expressed as absolute numbers (percentage) or as median values [25–75 interquartile range (IQR)]. Categorical variables were compared using the chi-square test or Fisher’s exact test. The Mann–Whitney U test was used to compare quantitative variables from two independent groups. For comparison of three or more independent groups, Kruskal-Wallis test was used. Friedman’s or Wilcoxon’s rank tests were used to perform paired analysis of variables. A two-tailed *p* value of <0.05 was considered to be significant. The association between quantitative variables was analyzed using the Spearman correlation test. In order to reduce the possibility of correlations due to chance, an absolute Spearman correlation coefficient (|ρ|) ≥ 0.6 was established as significant. SPSS 22.0 statistical software package (SPSS Inc., Chicago, IL, United States) was used to perform statistical analysis.

The raw sequencing data were in FASTQ format. Quality analysis of reads from Miseq Illumina was performed with the FastQC program (Andrews, 2010). Sequences were analyzed with the QIIME2 software package, version 2020.11 (Caporaso et al., 2010). Using the DADA2 tool (Callahan et al., 2016), low-quality regions were truncated, sequences were assembled, noise was corrected and removed, and sequences were grouped by ASVs (amplicon sequence variants). The 16S rRNA representative sequence was constructed using the silva-138-99 (https://www.arb-silva.de; Silva) amplicon database with a similarity threshold of 97%, using the QIIME2 platform. The filtered sequences were aligned and grouped into ASV-based sequence clusters. Thus, the taxonomic analysis of the sequences was obtained, and the sequences were collapsed (clustered) to the genus level.

Fecal microbiota analysis was performed using R software version 4.3.3. To minimize biases due to uneven sequencing depth, sequence counts were rarefied to 90% of the minimum sequencing depth across samples. Alpha diversity was calculated using the *phyloseq* package and the rarified data, using three different measures: Observed species, the Chao1 index, and the Shannon index, visualized by the *stat* and *ggplot2* packages. Statistical significance between groups of alpha diversity was assessed using Wilcoxon-Mann-Whitney or Kruskal-Wallis test. Beta diversity was analyzed with the adonis2 function and the rarified data, and for the principal coordinate analysis (PCoA), the distance matrices were calculated using the weighted UniFrac distance, using the *vegan* and *microbiome* packages. It was visualized using the *ggplot2* package in R 4.3.3.

For the selection of the most abundant genera, a *phyloseq* object was used with the rarified ASVs abundance data, selecting the genera with the highest median abundance of each study group with the functions of the *dplyr* package. Subsequently, using the *ggplot2* library, a stacked bar graph was generated for each group showing the mean abundance of the selected genera.

The analysis of significant differences in taxonomic abundance was carried out using the LEfSe (Linear Discriminant Analysis Effect Size) method (Segata et al., 2011), at the genus level. It was performed using the *run_lefse* function of the *microbiome* package, with the study group as the grouping variable and normalizing the data using the CPM (counts per million) method. LEfSe applies a Kruskal-Wallis test for initial group screening and Wilcoxon rank-sum tests for pairwise comparisons within its standard workflow. Groups with p < 0.05 (using the Wilcoxon test) were considered significant, establishing a different LDA score cut-off point depending on the number of genera obtained in each comparison. Correlations at baseline (t0) were visualized as a heatmap using Morpheus (https://software.broadinstitute.org/morpheus/), including only those microbial genera deemed significant by LEfSe.

## Results

The demographic, immunological, and virological characteristics, along with gut barrier status, bacterial translocation of PLWH and controls, are shown in the **Table 1**. There were no statistically significant differences in age and sex between PLWH and controls.

**Table 1:** Demographic, gut barrier, bacterial translocation, immunological, and virological profiles in people living with HIV (PLWH) at baseline (t0), after six months of ART (t6), and in HIV-uninfected controls.

Variable	Controls (n=18) (1)	PLWH t=0 (n=17) (2)	PLWH t=6m (n=17) (3)	p (1 vs 2)	p (1 vs 3)	p (2 vs 3)
Demographic characteristics						
Age (years)	39 (33-43)	40 (34-50)		0.372		
Sex male (n, %)	15 (83)	16 (94)		0.603		
Virological characteristics						
Viral load (copies/ml)		118400 (22250-491000)	0 (0-0)			**< 0.001**
Gut barrier damage and bacterial translocation						
I-FABP (ng/ml)	767 (574-902)	950 (800-1168)	1049 (829-1367)	**0.012**	**0.003**	0.687
16S rDNA (copies/ml)	2162 (2023-2286)	4281 (4018-4485)	2878 (2761-3277)	**< 0.001**	**0.002**	0.068
Systemic inflammatory parameters						
IL-6 (pg/ml)	0.8 (0.3-1.3)	4.8 (3.1-8.8)	3.3 (1.7-4.0)	**< 0.001**	**< 0.001**	**0.022**
sCD163 (ng/ml)	561 (425-662)	1173 (872-1417)	795 (549-915)	**< 0.001**	**0.006**	**< 0.001**
Anti-inflammatory molecule						
IL-10 (pg/ml)	1.5 (0.0-2.3)	6.6 (3.0-9.2)	2.5 (1.3-5.2)	**< 0.001**	**0.012**	0.121
Lymphocyte number and percentage and lymphocyte activation						
Lymphocytes/mm^3^	1760 (1375-2435)	2375 (1845-2805)	2195 (1528-2990)	**0.042**	0.361	0.084
Absolute CD4^+^ T cells (cells/mm^3^)	886 (513-1119)	600 (278-807)	747 (461-1141)	**0.011**	0.547	**0.001**
CD4^+^ T cells (% of lymphocytes)	50.80 (43.82-61.53)	21.60 (16.25-33.85)	32.00 (24.30-38.35)	**< 0.001**	**< 0.001**	**0.007**
Absolute CD8^+^ T cells (cells/mm^3^)	579 (405-791)	1084 (898-1386)	1000 (815-1360)	**< 0.001**	**< 0.001**	0.394
CD8 T cells (% of lymphocytes)	31.80 (27.08-34.93)	55.10 (41.20-64.10)	47.00 (35.60-56.00)	**< 0.001**	**< 0.001**	**0.019**
CD4^+^CD38^+^HLA-DR^+^ (% of CD4^+^ T lymphocytes)	1.3 (0.3-2.2)	9.7 (2.8-18.5)	2.7 (1.7-6.3)	**0.007**	**0.018**	**0.047**
CD8^+^CD38^+^HLA-DR^+^ (% of CD8^+^ T lymphocytes)	1.7 (0.6-5.7)	19.0 (12.0-31.1)	6.5 (3.7-15.5)	**0.005**	**0.008**	0.086

Data are provided as absolute number (percentage) or as median (interquartile range). The Mann–Whitney U test was used to compare quantitative variables from two independent groups.

CD4+CD38+HLA-DR+, activated CD4+ T cells; CD8+CD38+HLA-DR+, activated CD8+ T cells; I-FABP, intestinal fatty acid binding protein; IL-6, interleukin 6; IL-10, interleukin 10; sCD163, soluble CD163. Comparisons with statistically significant differences are indicated in bold.

### Fecal microbiota characterization of study participants

**Figure 1:**
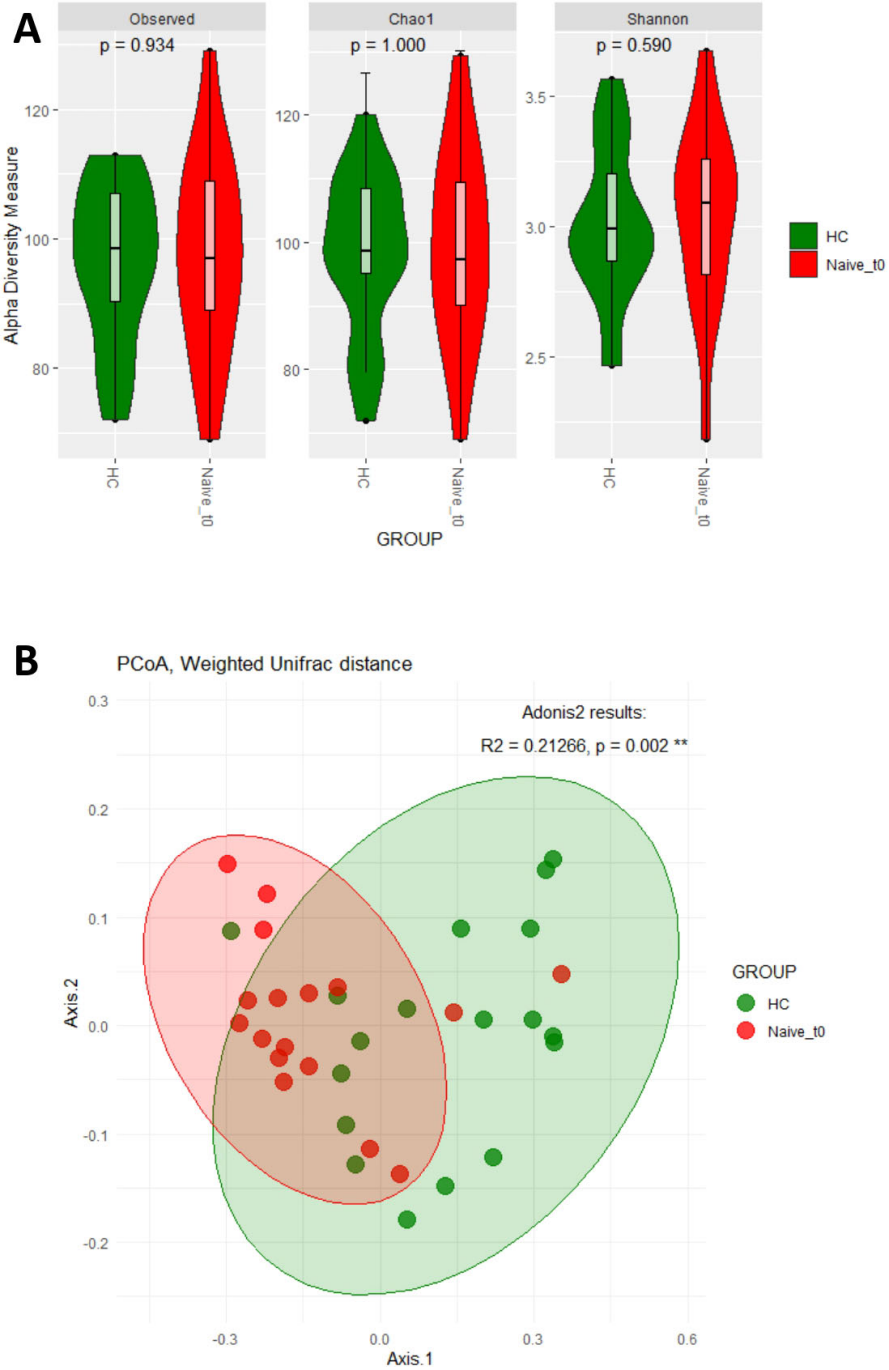
Alpha and beta diversity of fecal microbiota in PLWH at baseline and healthy controls (HC). **(A)** Alpha diversity measured by Observed species, Chao1, and Shannon indices, compared between groups using the Wilcoxon rank-sum test. **(B)** Beta diversity visualized by Principal Coordinates Analysis (PCoA) based on Weighted UniFrac distance. Color coding: controls (HC, green), naive PLWH at baseline (Naïve_t0, red).

**Figure 2:**
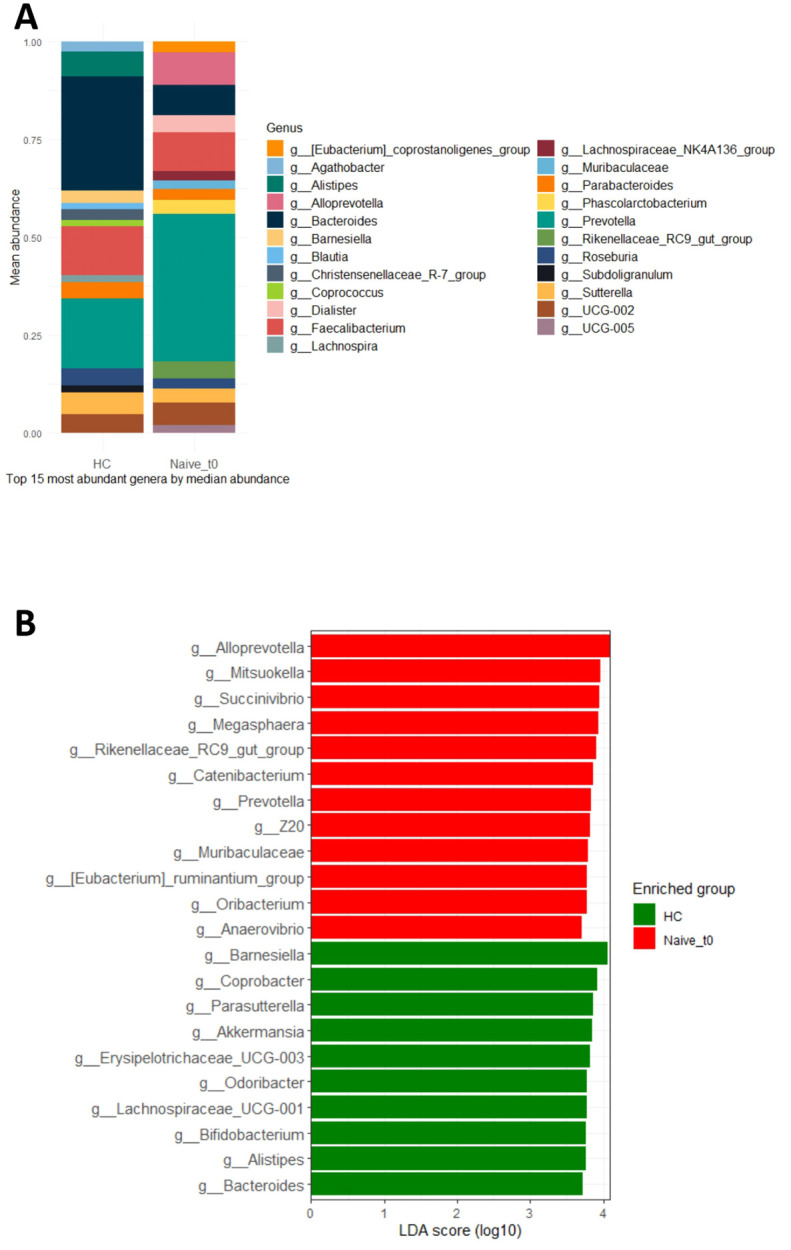
Taxonomic composition and LEfSe analysis comparing PLWH at baseline (t0) and healthy controls (HC). **(A)** Relative abundance of the 15 most abundant genera across groups (selected based on median abundance and displayed as mean relative abundance for visualization). **(B)** LEfSe (Linear discriminant analysis Effect Size) analysis showing genera significantly enriched with Linear Discriminant Analysis (LDA) score cutoff of 3.7 (log10). Color coding: HC (green), naive PLWH at baseline (Naïve_t0, red). Note: genera labeled as *g:UCG-002* and *g:UCG-005* correspond to unclassified members of the family *Oscillospiraceae*.

While alpha diversity was similar between untreated PLWH (t0) and controls (**Figure 1A**), beta diversity differed significantly between the groups (**Figure 1B**), revealing a clear separation in microbial community structure. Examination of the 15 most abundant genera revealed that *Bacteroides* was the predominant genus in controls, while *Prevotella* was the most abundant in untreated PLWH, and ranked second in controls. *Faecalibacterium* was notably abundant in both groups (**Figure 2A**). LEfSe analysis of fecal microbiota composition identified *Alloprevotella*, *Mitsuokella*, *Succinivibrio*, and *Megasphaera* as the genera most enriched in untreated PLWH compared to controls. Conversely, *Barnesiella*, *Coprobacter*, *Parasutterella*, and *Akkermansia* were the genera most enriched in controls. *Bacteroides* was also enriched in this group (**Figure 2B**).

**Figure 3:**
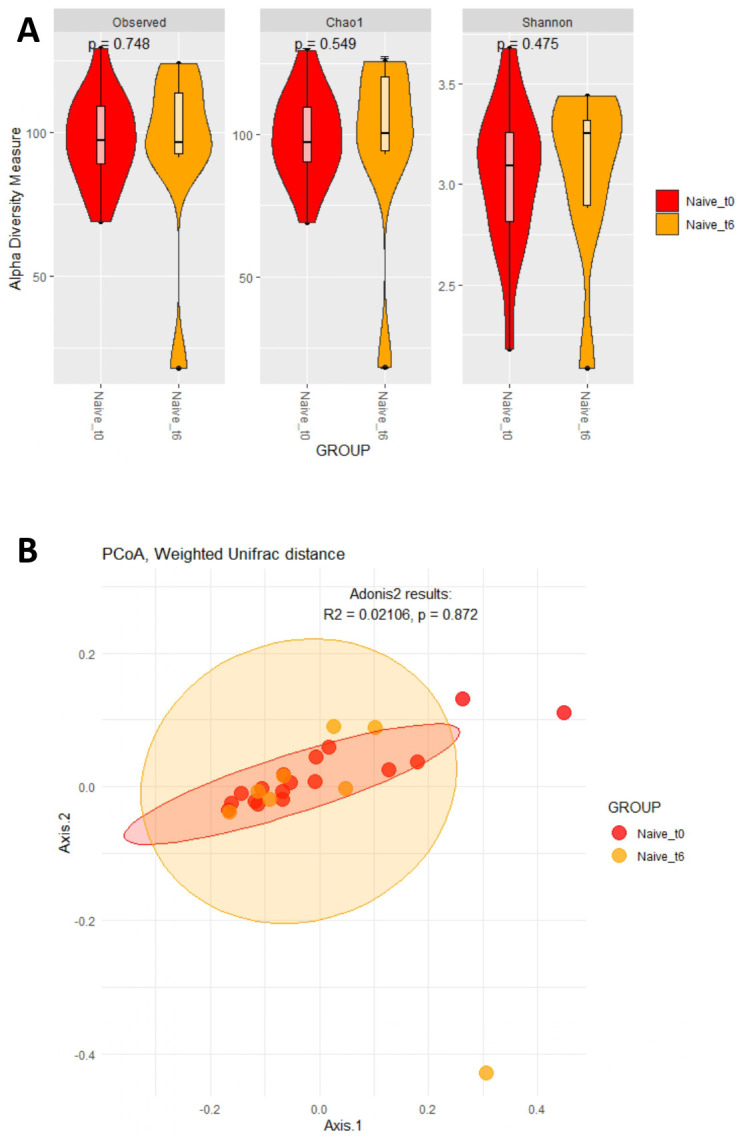
Alpha and beta diversity of fecal microbiota in PLWH at baseline (Naïve_t0) and six months post-ART (Naïve_t6). **(A)** Alpha diversity measured by Observed species, Chao1, and Shannon indices, compared between groups using the Wilcoxon rank-sum test. **(B)** Beta diversity visualized by Principal Coordinates Analysis (PCoA) based on Weighted UniFrac distance. Color coding: naive PLWH at baseline (Naïve_t0, red), naive PLWH at six months post-ART (Naïve_t6, orange).

**Figure 4:**
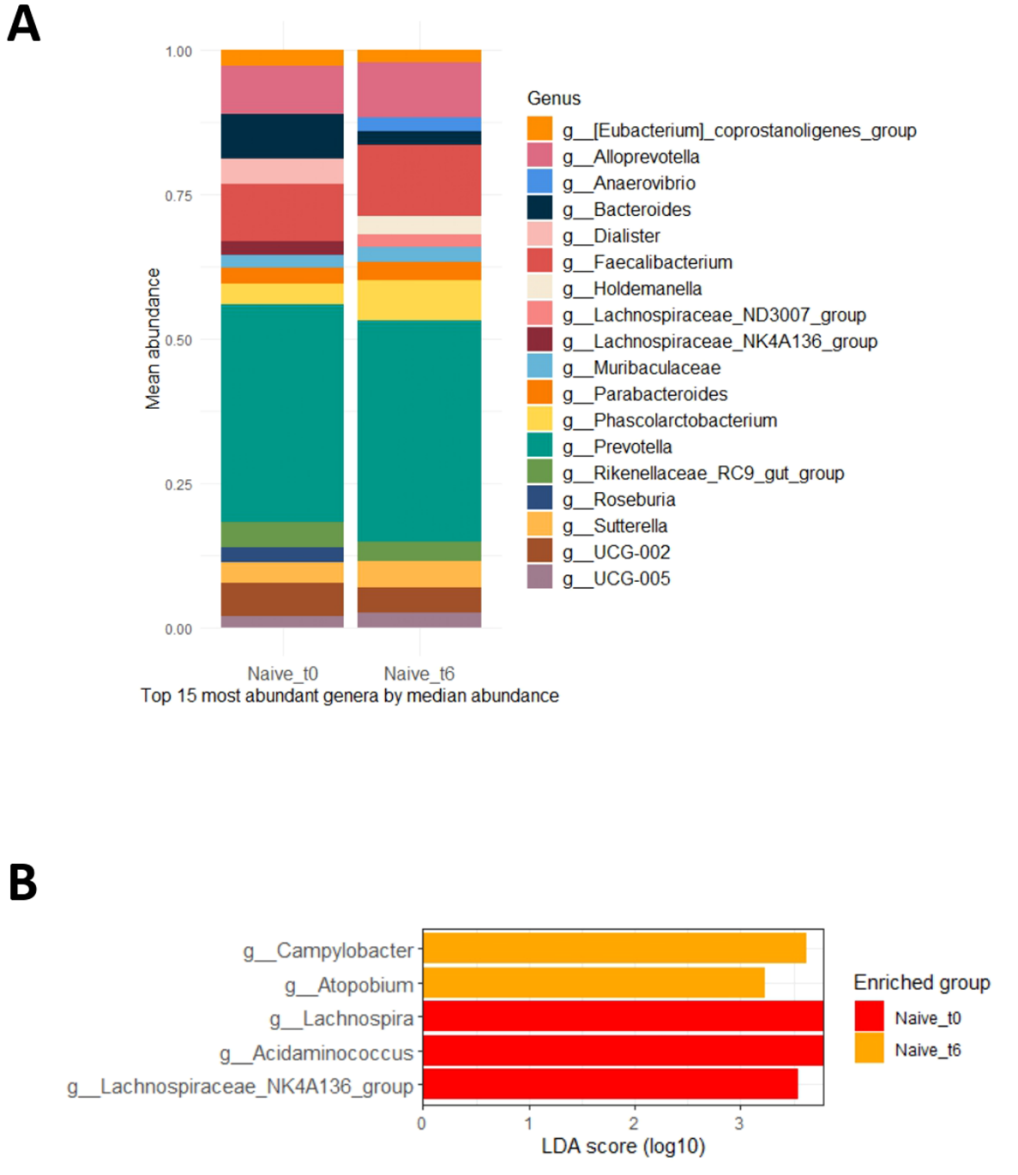
Taxonomic composition and LEfSe analysis comparing PLWH at baseline (t0) and six months post-ART (t6). **(A)** Relative abundance of the 15 most abundant genera across groups (selected based on median abundance and displayed as mean relative abundance for visualization). **(B)** LEfSe (Linear discriminant analysis Effect Size) analysis showing genera significantly enriched with Linear Discriminant Analysis (LDA) score cutoff of 3 (log10). Color coding: naive PLWH at baseline (Naïve_t0, red), naive PLWH at six months post-ART (Naïve_t6, orange). genera labeled as *g:UCG-002* and *g:UCG-005* correspond to unclassified members of the family *Oscillospiraceae*.

The initiation of ART and subsequent viral suppression did not induce a major shift in the gut microbiota. Both alpha and beta diversity remained similar between PLWH at t0 and t6 (**Figures 3A, B**), indicating that six months of therapy is insufficient to induce a deep structural remodeling of the microbiome. The global composition of the predominant genera remained unchanged (**Figure 4A**), with *Prevotella*, *Faecalibacterium*, and *Alloprevotella* as the predominant genera at both t0 and t6. LEfSe analysis identified significant shifts in the abundance of certain taxa (**Figure 4B**). An enrichment of *Campylobacter* and *Atopobium* was observed at t6. Compared to treated PLWH (t6), the genera *Lachnospira*, *Acidaminococcus*, and *Lachnospiraceae NK4A136* group were enriched at t0.

**Figure 5:**
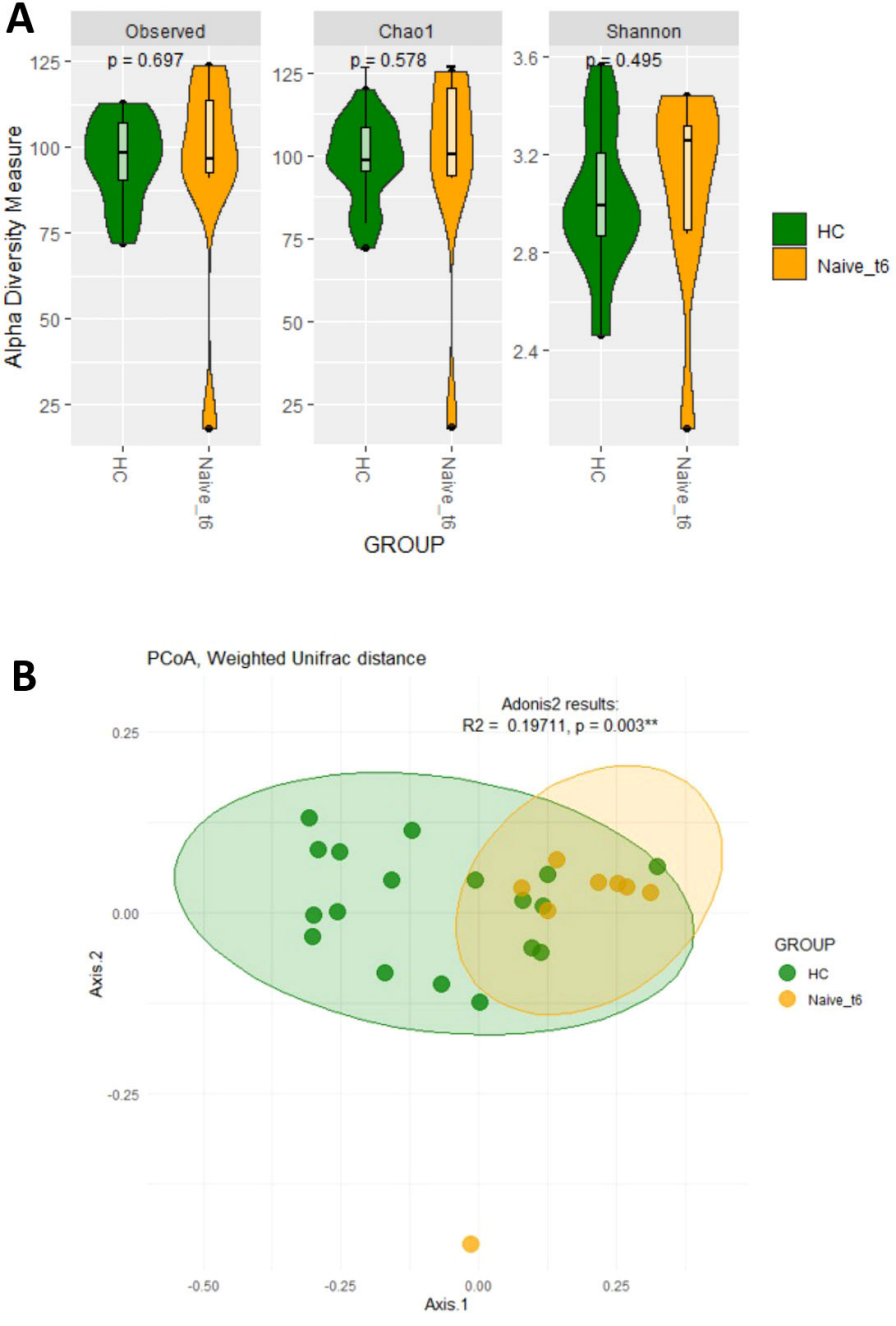
Alpha and beta diversity of fecal microbiota in PLWH six months post-ART (Naïve_t6) and healthy controls (HC). **(A)** Alpha diversity measured by Observed species, Chao1, and Shannon indices, compared between groups using the Wilcoxon rank-sum test. **(B)** Beta diversity visualized by Principal Coordinates Analysis (PCoA) based on Weighted UniFrac distance. Color coding: controls (HC, green), naive PLWH at six months post-ART (Naïve_t6, orange).

**Figure 6:**
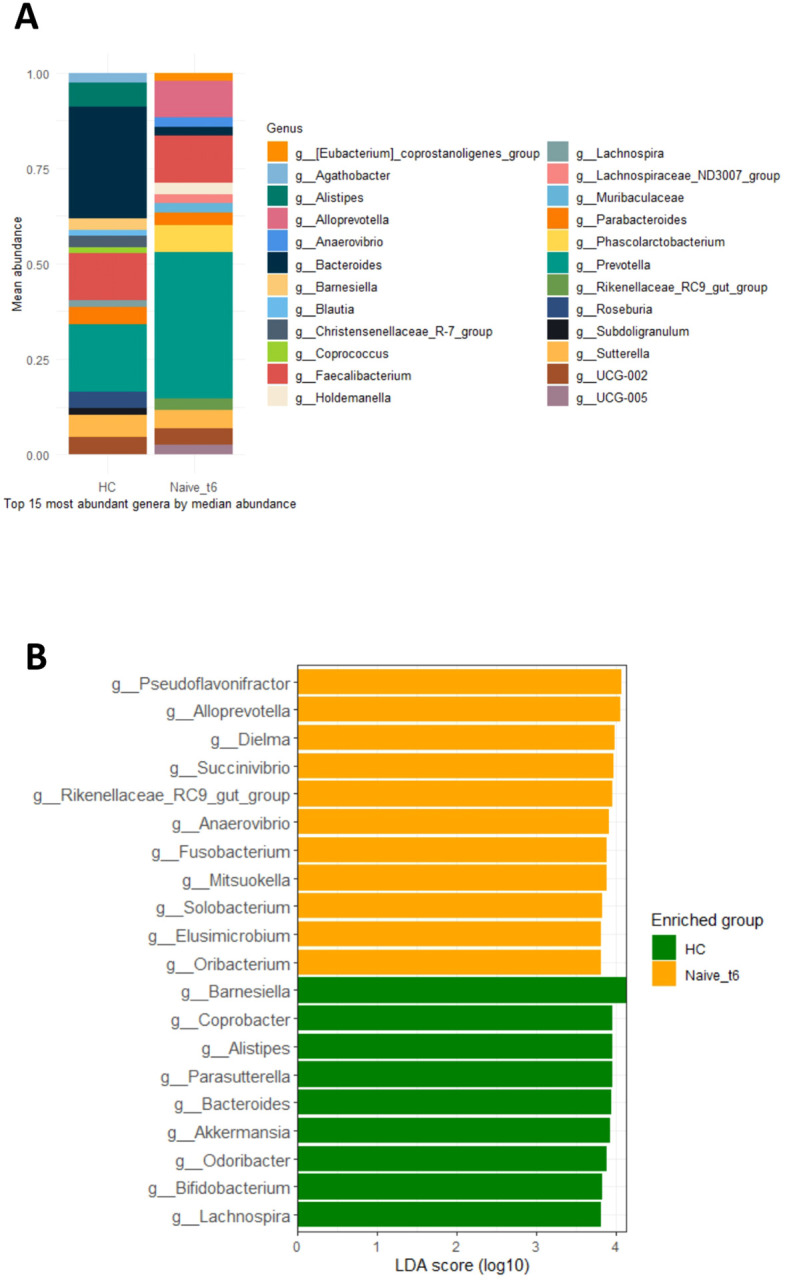
Taxonomic composition and LEfSe analysis comparing PLWH six months post-ART (Naïve_t6) and healthy controls (HC). **(A)** Relative abundance of the 15 most abundant genera across groups. **(B)** LEfSe (Linear discriminant analysis Effect Size) analysis showing genera significantly enriched with Linear Discriminant Analysis (LDA) score cutoff of 3 (log10). Color coding: Color coding: controls (HC, green), naive PLWH at six months post-ART (Naïve_t6, orange).

In relation to the controls, PLWH after treatment (t6) showed similar alpha diversity but significant differences were detected regarding beta diversity (**Figures 5A, B**). The most abundant genera in PLWH at t6 were *Prevotella*, *Faecalibacterium*, *Alloprevotella*, and *Phascolarctobacterium*. LEfSe analysis identified *Pseudoflavonifractor, Alloprevotella*, *Dielma and Succinivibrio* and as the genera most enriched in treated PLWH compared to controls (**Figure 6**).

### Gut barrier integrity and bacterial translocation of study participants

Compared to controls, PLWH at t0 showed gut barrier damage [I-FABP, 950 (800-1168) vs 767 (574-902) ng/mL, p=0.012] and elevated bacterial translocation [16S rDNA, 4281 (4018-4485) vs 2162 (2023-2286) copies/mL, p<0.001]. After six months of ART (t6), I-FABP remain significantly increased compared to controls. A non-significant reduction in 16S rDNA values ​​of PLWH, measured at t0 and t6 [from 4281 (4018-4485) to 2878 (2761-3277) copies/ml, p=0.068] was observed, although significant differences with respect to controls persisted (**Table 1**).

### Systemic inflammatory parameters of study participants

Increased inflammatory markers such as IL-6 [4.8 (3.1-8.8) vs 0.8 (0.3-1.3) pg/mL, p<0.001], sCD163 [1173 (872-1417) vs 561 (425-662) ng/mL, p=<0.001], and the anti-inflammatory cytokine IL-10 [6.6 (3.0-9.2) vs 1.5 (0.0-2.3) pg/mL, p<0.001] were detected in untreated PLWH compared to controls.

After six months of ART (t6), markers of inflammation (IL-6 and sCD163) were significantly decreased [IL-6, 3.3 (1.7-4.0) vs 4.8 (3.1-8.8) pg/mL, p=0.022; sCD163, 795 (549-915) vs 1173 (872-1417) ng/mL, p<0.001].

Compared to controls, PLWH at t6 continued to show significantly increased serum concentrations of IL-6, sCD163, and IL-10 (**Table 1**).

### CD4^+^ and CD8^+^ T lymphocyte numbers and lymphocyte activation of study participants

CD4^+^ T cell counts were reduced [600 (278-807) vs 886 (513-1119) cells/mm3, p=0.011] and activated CD4^+^ [9.7 (2.8-18.5) vs 1.3 (0.3-2.2), percentage of absolute CD4+ T cells, p=0.007] and CD8^+^ T cells [19.0 (12.0-31.1) vs 1.7 (0.6-5.7), percentage of absolute CD8+ T cells, p=0.005] were increased in PLWH at t0 compared to controls.

After six months of ART (t6), PLWH showed improved immune status characterized by increased CD4^+^ T cell counts compared to untreated PLWH [747 (461-1141) vs 600 (278-807) cells/mm3, p=0.001]. A significant decrease in the percentage of activated CD4^+^ T lymphocytes [2.7 (1.7-6.3) vs 9.7 (2.8-18.5), percentage of absolute CD4+ T cells, p=0.047], but not of CD8^+^ T lymphocytes [6.5 (3.7-15.5) vs 19.0 (12.0-31.1), percentage of absolute CD8+ T cells, p=0.086], was observed.

Compared to controls, PLWH at t6 continued to show significantly increased percentages of activated CD4^+^ and CD8^+^ T lymphocytes (**Table 1**).

### Correlations between microbiota and immune/inflammatory parameters

**Figure 7:**
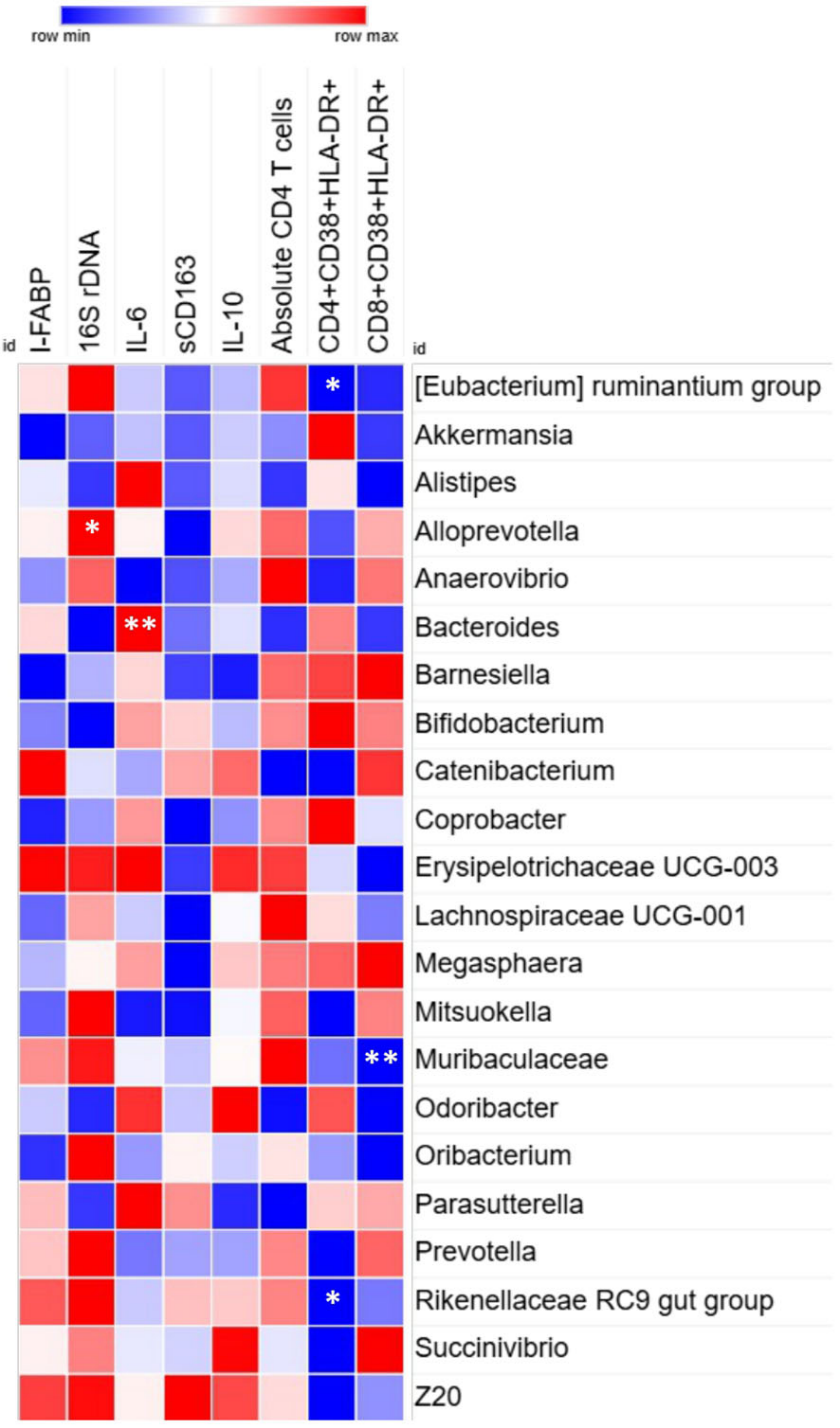
Heatmap of Spearman correlations between bacterial genera significantly enriched by LEfSe at baseline (t0) and immunological/inflammatory parameters in PLWH. Correlations are represented by color intensity (*p < 0.05, **p < 0.01). Positive correlations in red, negative correlations in blue. 16S rDNA, 16S ribosomal DNA; CD4+CD38+HLA-DR+, activated CD4+ T cells; CD8+CD38+HLA-DR+, activated CD8+ T cells; I-FABP, intestinal fatty acid binding protein; IL-6, interleukin-6; IL-10, interleukin-10; sCD163, soluble CD163.

Several significant correlations (|ρ| ≥ 0.6, p < 0.05) between LEfSe-identified bacterial genera and immunological/inflammatory parameters were observed in PLWH at t0 (**Figure 7**; **Supplementary Table 1**). Notable findings included negative associations of *[Eubacterium] ruminantium* group and *Rikenellaceae RC9* gut group with activated CD4^+^ T cells, *Muribaculaceae* with activated CD8^+^ T cells, and positive correlations of *Alloprevotella* with bacterial translocation (16S rDNA) and *Bacteroides* with IL-6.

**Figure 8:**
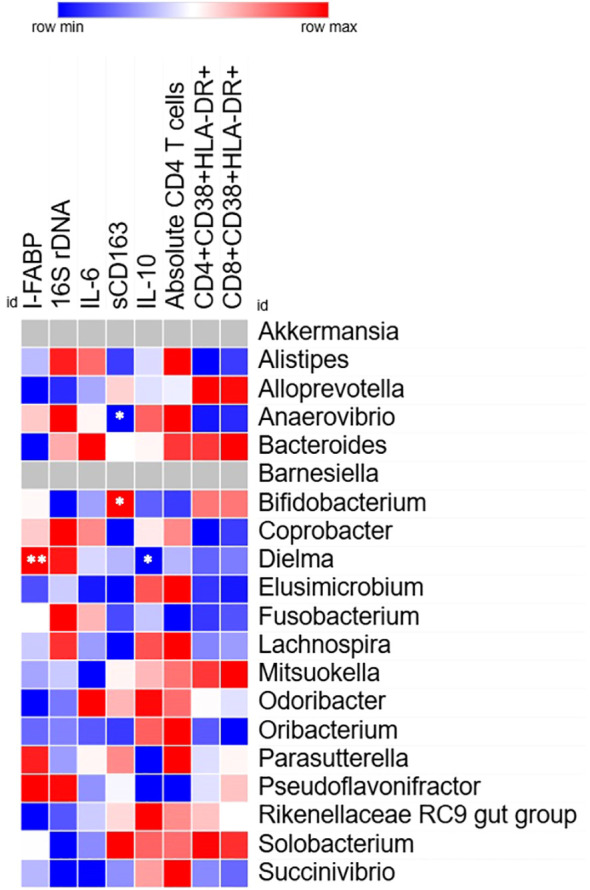
Heatmap of Spearman correlations between bacterial genera significantly enriched by LEfSe at 6 months after ART initiation (t6) and immunological/inflammatory parameters in PLWH. Correlations are represented by color intensity (*p < 0.05, **p < 0.01). Positive correlations in red, negative correlations in blue. 16S rDNA, 16S ribosomal DNA; CD4+CD38+HLA-DR+, activated CD4+ T cells; CD8+CD38+HLA-DR+, activated CD8+ T cells; I-FABP, intestinal fatty acid binding protein; IL-6, interleukin-6; IL-10, interleukin-10; sCD163, soluble CD163. Gray rows indicate genera for which no bacterial DNA copies were detected in any of the samples analyzed in the t6 group.

At t6, several significant correlations were also identified between LEfSe-identified bacterial genera and immunological/inflammatory markers (**Figure 8**; **Supplementary Table 2**). Specifically, *Anaerovibrio* showed a negative correlation with sCD163, while *Bifidobacterium* correlated positively with sCD163. *Dielma* was negatively associated with IL-10 and positively associated with I-FABP.

## Discussion

Our study reinforces and extends previous observations reported by our group and others regarding gut barrier dysfunction, increased bacterial translocation, and systemic immune and inflammatory activation in PLWH [Jiang et al., 2009; Cuesta-Sancho et al., 2023; Illanes-Álvarez et al., 2026). Consistent with those prior data, we found that these abnormalities improve after initiation of ART and achievement of an undetectable viral load. However, despite this improvement, a state of persistent immune activation and inflammation remains detectable. This underscores the complex and chronic nature of immune dysregulation in HIV even under effective viral suppression.

Our analysis highlights distinct differences in the fecal microbiota between PLWH at baseline (t0) and HIV-uninfected controls. Interestingly, LEfSe analysis revealed that *Alloprevotella*, *Mitsuokella*, *Succinivibrio*, and *Megasphaera* were among the genera most enriched in PLWH at t0. While these genera are generally considered beneficial in gut health due to the production of SCFAs (Louis and Flint, 2009; Downes et al., 2013; Willems and Collins, 2015; Ticinesi et al., 2017; Parada Venegas et al., 2019; Lin et al., 2021), and key metabolites for maintaining intestinal homeostasis and modulating immune function (den Besten et al., 2013; Lozupone et al., 2013; Mathewson et al., 2016; Yang et al., 2020), their increase in the context of untreated HIV raises intriguing questions about their specific functional roles or strain-level variations. The key question becomes: what ecological forces in the damaged gut could favor the modification of the microbiota? One speculative model is the following: 1) HIV damages the intestinal epithelium (Brenchley et al., 2006). 2) The composition and renewal of the mucus layer can be altered, and epithelial injury can increase the release of glycans and mucins (Sandler and Douek, 2012). These host-derived carbohydrates can serve as substrates for certain intestinal microbes, particularly mucin-degrading bacteria (*Akkermansia muciniphila* or *Bacteroides* spp.), releasing sugars such as fucose, galactose, N-acetylglucosamine, and sialic acid (Tiffany et al., 2026). 3) Although secondary fermenters (such as SCFA-producing bacteria) cannot break down intact mucin on their own, they feed on these released sugars (cross-feeding), converting them into vital SCFAs, such as butyrate, acetate, and propionate (Raimondi et al., 2021; Tiffany et al., 2026) and expand (Flint et al., 2012).

Controls showed enrichment mainly of *Barnesiella*, *Coprobacter*, *Parasutterella* and *Akkermansia* compared with PLWH at t0, with *Bacteroides* also notably enriched in the control group. These genera are recognized for their beneficial roles in maintaining gut barrier integrity and modulating immune responses (Ubeda et al., 2013; Shkoporov et al., 2013; Lukovac et al., 2014; Ju et al., 2019; Zafar and Saier, 2021; Xia et al., 2022). For example, *Akkermansia* contributes to gut barrier protection by stimulating mucin production, thereby supporting the integrity of enterocytes, and it has been consistently reported to be decreased in PLWH (Rocafort et al., 2019; Zhao et al., 2023). *Parasutterella* is involved in the metabolism of tryptophan, producing metabolites that influence immune regulation and may contribute to maintaining mucosal homeostasis (Ju et al., 2019). Taken together, our data suggest that dysbiosis in PLWH is not primarily characterized by a decrease in SCFA-producing genera, but rather by a reduction in bacteria that support the intestinal barrier through alternative mechanisms. It is therefore conceivable that bacterial translocation largely reflects the passage of specific bacterial products that, although not dominant in the fecal microbiota, have an increased ability to cross the compromised intestinal barrier. The enrichment of some of these genera in the intestinal mucosa, such as those belonging to the phylum *Proteobacteria*, has been correlated with the presence of systemic endotoxemia (Dillon et al., 2014). This finding has also been supported by other authors who have demonstrated a correlation between *Enterobacteriaceae* enrichment and systemic inflammatory activation (Dinh et al., 2015).

The correlation analyses offered valuable insights into the complex interplay between the gut microbiota and the host immune system in PLWH at baseline. Several genera exhibited negative correlations with immune activation markers, suggesting potential protective roles. *Rikenellaceae RC9* gut group, an SCFA producer (Graf, 2014), as well as *[Eubacterium] ruminantium* group, both enriched in PLWH, negatively correlated with activated CD4^+^ T lymphocytes, potentially reflecting their role in dampening CD4^+^ T cell activation. Another SCFA-producing taxa, *Muribaculaceae* (He et al., 2022) —a family that also produces mucus supporting gut barrier integrity— demonstrated a negative correlation with activated CD8^+^ T lymphocytes, reinforcing its putative anti-inflammatory functions. Conversely, positive correlations were observed between *Bacteroides* (enriched in controls) and IL-6, and between *Alloprevotella* (enriched in PLWH) and bacterial translocation (16S rDNA). The *Bacteroides*-IL-6 correlation is intriguing, as *Bacteroides* is typically viewed as anti-inflammatory while IL-6 is pro-inflammatory. This suggests that *Bacteroides* supports physiologic immune homeostasis rather than pathology —specifically, a controlled pro-inflammatory state maintained through IL-6-mediated mechanisms such as intraepithelial lymphocyte activation and epithelial barrier reinforcement (Kuhn et al., 2018). Remarkably, *Alloprevotella* expansion has also been observed in ulcerative colitis mouse models (Wang et al., 2018; Huang et al., 2022) —a condition characterized by bacterial translocation akin to HIV infection (Gutiérrez et al., 2009; Johansson et al., 2014) — where its abundance decreases following effective treatment (Huang et al., 2022), further supporting its association with compromised barrier function and microbial leakage. Although below our |ρ| ≥ 0.6 cutoff, the positive correlation between the abundance of *Succinivibrio* and the serum IL-10 (anti-inflammatory cytokine) (ρ=0.496, p=0.043) is also noteworthy. Notably, no significant correlations were observed between absolute CD4^+^ T cell counts and any bacterial genera.

An important finding of this longitudinal study is the stability of the gut microbiota despite viral suppression. After 6 months of ART both alpha and beta diversity remained similar to baseline levels. Moreover, LEfSe analysis at t6 identified *Campylobacter* and *Atopobium* as the only genera enriched compared to baseline in PLWH. Both genera are considered detrimental: *Atopobium* has been linked to colorectal cancer (Yachida et al., 2019), while *Campylobacter* is a well-known cause of gastroenteritis and can provoke systemic complications, especially in immunocompromised individuals (Costa and Iraola, 2019). In contrast, at t0, the genera *Lachnospira*, *Acidaminococcus*, and *Lachnospiraceae NK4A136* group were enriched. These three taxa are producers of SCFAs (García-Sánchez et al., 2010; Ma et al., 2020; Yu et al., 2022). The presence of beneficial SCFA-producing bacteria before ART initiation, followed by enrichment of pathogenic taxa at t6, was unexpected and may represent ART side effects or a compensatory response whereby the body increased SCFA producers prior to treatment to counteract inflammation, as previously mentioned.

Despite these shifts, changes in the gut microbiota over six months are modest. Compared to controls, the fecal microbiota of PLWH after 6 months of ART (t6) continued to show differences characterized by a predominance of *Alloprevotella* and *Succinivibrio*, among others. These genera, which are considered beneficial due to their SCFA production, further support the idea that their enrichment may represent a response to intestinal barrier injury, as previously discussed, and this pattern was observed in PLWH and persisted after ART. Other genera, such as *Pseudoflavonifractor* and *Dielma*, were also enriched in PLWH after ART. Members of the genus *Pseudoflavonifractor* are capable of weakly fermenting some sugars and produce acetic and succinic acids (Alauzet et al., 2023). *Dielma* is a genus belonging to the family *Erysipelotrichaceae*, associated with certain protective markers, including an anti-inflammatory effect (Nakajima et al., 2022). The positive correlation between the abundance of this genus and serum I-FABP concentration, as well as its negative correlation with IL-10 levels, may seem paradoxical. We propose that it reflects an expansion of *Dielma* as a compensatory response to intestinal injury and local inflammation. A similar explanation may apply to the positive correlation between the abundance of another beneficial genus, *Bifidobacterium* (O'Callaghan and van Sinderen, 2016), and serum sCD163 concentration. Likewise, we propose that the negative correlation between sCD163 and the abundance of *Anaerovibrio* would be related to the lower availability of lipid substrates after the inflammatory response, since *Anaerovibrio* has been implicated in lipid hydrolysis (Privé et al., 2013).

It is remarkable that PLWH with long-term viral suppression (median 121 months) exhibit persistent gut microbiota alterations (similar to those described in the present article) despite comparable alpha/beta diversity to controls, characterized by enrichment of *Succinivibrio*, *Megasphaera*, *Alloprevotella*, and *Faecalitalea* (all SCFA-producers) alongside reduced *Akkermansia* and *Paraprevotella* —bacteria implicated in barrier integrity—compared to controls (Illanes-Álvarez et al., 2026), suggesting a specific gut microbiota signature in PLWH regardless of viral suppression status. Given that HIV-related intestinal lesions occur as early as the first 14 days of infection (Brenchley et al., 2006; Sandler and Douek, 2012), it is plausible that the adaptation of the gut microbiota was similarly prompt.

These persistent microbial alterations likely could be involved in the continued elevations in intestinal injury (I-FABP), bacterial translocation (16S rDNA), and inflammatory/immune activation markers observed despite ART, underscoring the incomplete normalization of gut-immune homeostasis in PLWH. At the same time, our longitudinal data at t6 provide crucial insights into the short-term kinetics of early antiretroviral intervention, demonstrating that ART produced a partial improvement in host inflammatory and immune markers. Specifically, serum concentrations of IL-6 and sCD163, alongside the percentage of activated CD4^+^ T lymphocytes, decreased significantly after six months of treatment. Despite the observed mitigations, none of these inflammatory or adaptive immune parameters achieved full normalization, remaining significantly elevated at t6 when compared directly to HIV-uninfected healthy controls. This sustained systemic inflammation closely mirrors the lack of clearance in bacterial translocation and the mucosal damage (I-FABP) captured at t6. Together, these findings indicate that while suppressive ART reduces acute systemic inflammatory pressure, it fails to fully reverse the underlying intestinal and immunological abnormalities established during early HIV infection, leaving the gut-immune axis in a state of chronic, low-grade activation.

This study has some limitations. First, more advanced longitudinal and stratified approaches, including potential confounding factors (such as age or sexual practices), could provide additional information. However, given the sample size of the present study, we believe that introducing more complex models would risk overfitting and would limit the robustness and interpretability of the findings. Second, the inherent limitations of correlation methods may prevent conclusions regarding causality. Future longitudinal studies with larger cohorts and extended follow-up periods will be essential to better understand the temporal dynamics and causal relationships between microbiota alterations and immune activation in HIV infection. Finally, direct measurement of SCFAs would have strengthened the mechanistic interpretation, provided in the “Discussion” section, used to explain the enrichment in genres that produce them. Unfortunately, SCFA quantification was not available in the current cohort.

## Conclusions

Our data confirm that the gut microbiota of PLWH differs markedly from that of HIV-uninfected individuals and is characterized by a paradoxical enrichment of SCFA-producing genera, likely useful as a compensatory response to counteract early intestinal barrier dysfunction and immune activation. Importantly, the six-month ART data show a dual outcome: while suppressive treatment achieved complete plasma viral clearance and attenuated systemic inflammation (IL-6, sCD163) along with CD4^+^ T-cell activation, underlying gut barrier damage and bacterial translocation persisted compared to controls. Furthermore, the control of viral replication is not accompanied by significant modifications of the intestinal microbiota, and dysbiosis persists. A better understanding of the gut microbiota is urgently needed to develop targeted adjunctive therapies to modulate this immune activation and improve long-term outcomes in PLWH.

## Data Availability

The datasets presented in this study can be found in online repositories. The names of the repository/repositories and accession number(s) can be found below: https://www.ebi.ac.uk/ena, PRJEB109299.
