# Supplementary material for: Effect of antiretroviral therapy on gut microbiota, immune activation, and inflammatory markers in newly diagnosed HIV patients
Source: Front Cell Infect Microbiol. 2026 Jul 27;16:1853799. doi: 10.3389/fcimb.2026.1853799 (PMC13453690; doi:10.3389/fcimb.2026.1853799)
Supplement: Supplementary file 1 [file SupplementaryFile1.docx]

Journal: Frontiers in Cellular and Infection Microbiology

Manuscript ID: 1853799

Supplementary material

**Effect of Antiretroviral Therapy on Gut Microbiota, Immune Activation, and Inflammatory Markers in Newly Diagnosed HIV Patients**

Denisse Márquez-Ruiz, Francisco Illanes-Álvarez, Andrés Martín-Aspas, Ismael Tinoco Racero, Irene Campaña-Gómez, Raquel Guerra-González, Alberto Romero-Palacios, Mercedes Márquez-Coello, Sara Cuesta-Sancho, José-Antonio Girón-González.

**Supplementary Table 1**. Spearman correlations between bacterial genera identified as significantly enriched by LEfSe at baseline (t0) and immunological and inflammatory parameters in PLWH, including only statistically significant correlations with |ρ| ≥ 0.6.

| **Bacterial genera and parameters correlated** | | **ρ** | **p-value** |
| --- | --- | --- | --- |
| *[Eubacterium] ruminantium* group | CD4^+^CD38^+^HLA-DR^+^ | -0.633 | 0.037 |
| *Alloprevotella* | 16S rDNA | 0.616 | 0.033 |
| *Bacteroides* | IL-6 | 0.682 | 0.003 |
| *Muribaculaceae* | CD8^+^CD38^+^HLA-DR^+^ | -0.802 | 0.005 |
| *Rikenellaceae RC9* gut group | CD4^+^CD38^+^HLA-DR^+^ | -0.656 | 0.028 |

ρ: Spearman's rank correlation coefficient; CD4^+^CD38^+^HLA-DR^+^: activated CD4^+^ T cells; CD8^+^CD38^+^HLA-DR^+^: activated CD4^+^ T cells; IL-6: interleukin 6.

**Supplementary Table 2**. Spearman correlations between bacterial genera identified as significantly enriched by LEfSe at six months after ART initiation (t6) and immunological and inflammatory parameters in PLWH, including only statistically significant correlations with |ρ| ≥ 0.6.

| **Bacterial genera and parameters correlated** | | **ρ** | **p-value** |
| --- | --- | --- | --- |
| *Anaerovibrio* | sCD163 | -0.736 | 0.038 |
| *Bifidobacterium* | sCD163 | 0.764 | 0.027 |
| *Dielma* | I-FABP | 0.856 | 0.007 |
| *Dielma* | IL-10 | -0.719 | 0.044 |

ρ: Spearman's rank correlation coefficient; I-FABP: intestinal fatty acid binding protein; IL-10: interleukin 10; sCD163: soluble CD163.
